# Author Correction: A mussel-inspired film for adhesion to wet buccal tissue and efficient buccal drug delivery

**DOI:** 10.1038/s41467-024-48275-4

**Published:** 2024-05-02

**Authors:** Shanshan Hu, Xibo Pei, Lunliang Duan, Zhou Zhu, Yanhua Liu, Junyu Chen, Tao Chen, Ping Ji, Qianbing Wan, Jian Wang

**Affiliations:** 1 State Key Laboratory of Oral Diseases, National Clinical Research Center for Oral Diseases, Department of Prosthodontics, West China Hospital of Stomatology, Sichuan University, Chengdu, China; 2 Chongqing Key Laboratory of Oral Diseases and Biomedical Sciences, Chongqing Municipal Key Laboratory of Oral Biomedical Engineering of Higher Education, Stomatological Hospital of Chongqing Medical University, Chongqing, China; 3 National Engineering Research Center for Inland Waterway Regulation, Chongqing Jiaotong University, Chongqing, China

Correction to: *Nature Communications* 10.1038/s41467-021-21989-5, published online 16 March 2021

The original version of this article contained errors in Figures 6c, 7b, and in Supplementary Figs. 5d, 8d, 8f, which occurred during the assembly of the figures due to similarity of the name of different groups.

In Figure 6c, the ‘2h PVA-DOPA6@PLGA-PVA NPs’ group was inadvertently duplicated from the ‘4h PVA-DOPA6@PLGA-PVA NPs’ group. In Figure 7b, the liver of ‘control’ group was a shifted field view of the image in the ‘PVA-DOPA4@PLGA-PDA NPs’ group. In Supplementary Fig. 5d, the ‘PLGA’ group was inadvertently duplicated from the ‘PLGA-PVA’ group. In Supplementary Figs. 8d, 8f, the ‘control’ group was a shifted field view of the image in the ‘PVA-DOPA4@PLGA-PDA NPs’ and ‘PVA-DOPA6@PLGA-PDA NPs’ groups, respectively. This has been corrected in both the PDF and HTML versions of the article.

### Supplementary information

Corrected Supplementary Information

