# Supplementary material for: A mussel-inspired film for adhesion to wet buccal tissue and efficient buccal drug delivery
Source: Nat Commun. 2021 Mar 16;12:1689. doi: 10.1038/s41467-021-21989-5 (PMC7966365; doi:10.1038/s41467-021-21989-5)
Supplement: Supplementary file 1 — Supplementary Information [file 41467_2021_21989_MOESM1_ESM.pdf]

Supplementary Information

A mussel-inspired film for adhesion to wet buccal tissue and efficient buccal drug delivery

Authors:

Shanshan Hu[1,2], Xibo Pei[1], Lunliang Duan[3], Zhou Zhu[1], Yanhua Liu[1], Junyu Chen[1], Tao Chen[2], Ping Ji[2], Qianbing Wan[1*] & Jian Wang[1*]

Affiliations:

[1]State Key Laboratory of Oral Diseases, National Clinical Research Center for Oral Diseases, Department of Prosthodontics, West China Hospital of Stomatology, Sichuan University, Chengdu, China. [2]Stomatological Hospital of Chongqing Medical University, Chongqing Key Laboratory of Oral Diseases and Biomedical Sciences, Chongqing Municipal Key Laboratory of Oral Biomedical Engineering of Higher Education, Chongqing, China. [3]National Engineering Research Center for Inland Waterway Regulation, Chongqing Jiaotong University, Chongqing, China.

These authors contributed equally: Shanshan Hu, Xibo Pei.

*Corresponding Authors. e-mail: champion@scu.edu.cn; ferowang@hotmail.com.

Supplementary figures

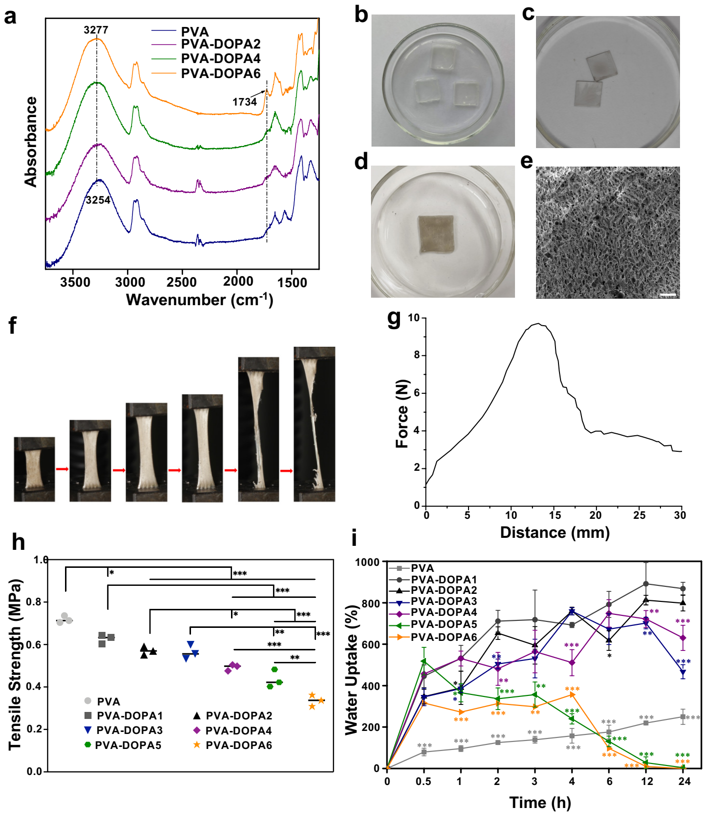

Supplementary Figure 1. Characterization of PVA-DOPA films. (a) FTIR spectra of PVA-DOPA polymers with different DOPA contents. PVA: poly(vinyl alcohol), DOPA: 3,4-dihydroxy-D-phenylalanine. (b) Photograph of ethyl cellulose protective cap. (c) Photograph of dry PVA-DOPA mucoadhesive film. (d) Photograph of PVA-DOPA mucoadhesive film after hydration. (e) SEM image of PVA-DOPA film. Scale bar: 200 μm (f) Tensile strength testing of PVA-DOPA3 film. (g) Representation of the stress-distance curve, during the tensile tests, for PVA-DOPA3 film. (h) Tensile strength of PVA-DOPA films with different DOPA contents. $n = 3$ independent samples per group; $^{*}P < 0.05$; $^{**}P < 0.01$; $^{***}P < 0.001$. (i) Swelling behavior of PVA-DOPA films different DOPA contents as a function of time. $n = 3$ independent samples per group; $^{*}P < 0.05$; $^{**}P < 0.01$; $^{***}P < 0.001$ vs PVA-DOPA1 group. All data are Mean ± S.D. Statistics was calculated by one-way ANOVA followed by Tukey's post-test. Exact P values are given in the Source Data file. Source data are provided as a Source data file.

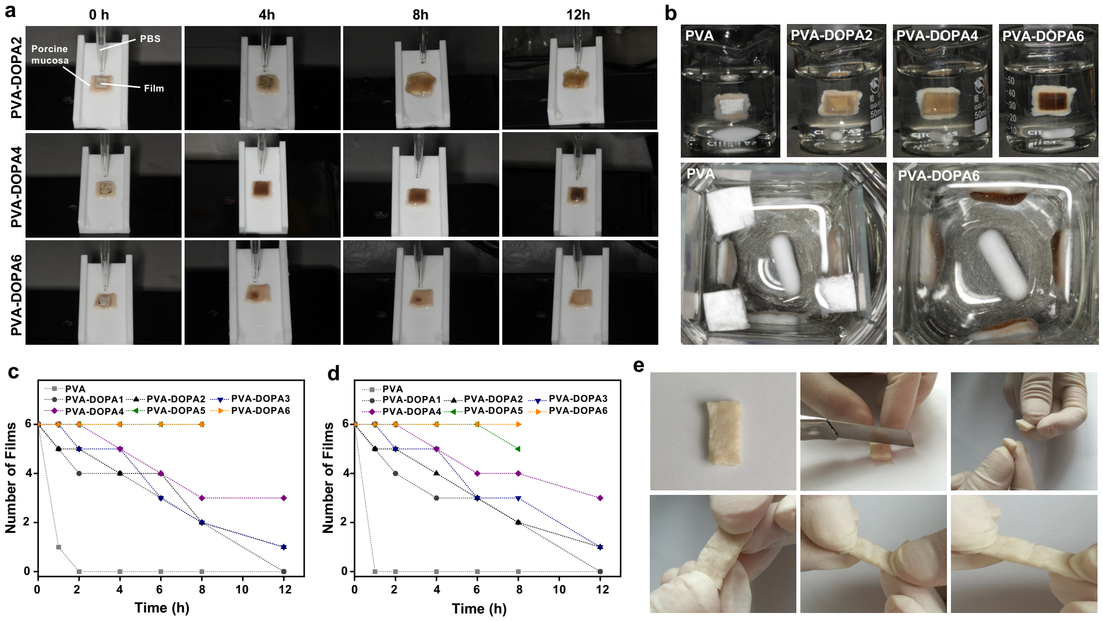

Supplementary Figure 2. Residence time and self-healing properties of PVA-DOPA films. (a) Experimental set-up for in vitro residence time measurement using the flow-through method. PVA: poly(vinyl alcohol), DOPA: 3,4-dihydroxy-D-phenylalanine. (b) Experimental set-up for in vitro residence time measurement using the rotating disc method. (c) The number of different PVA-DOPA films left on the porcine buccal mucosa as a function of time using the flow-through method. (d) The number of different PVA-DOPA films left on the porcine buccal mucosa as a function of time using the rotating disc method. (e) Photographs of self-healing properties of PVA-DOPA film.

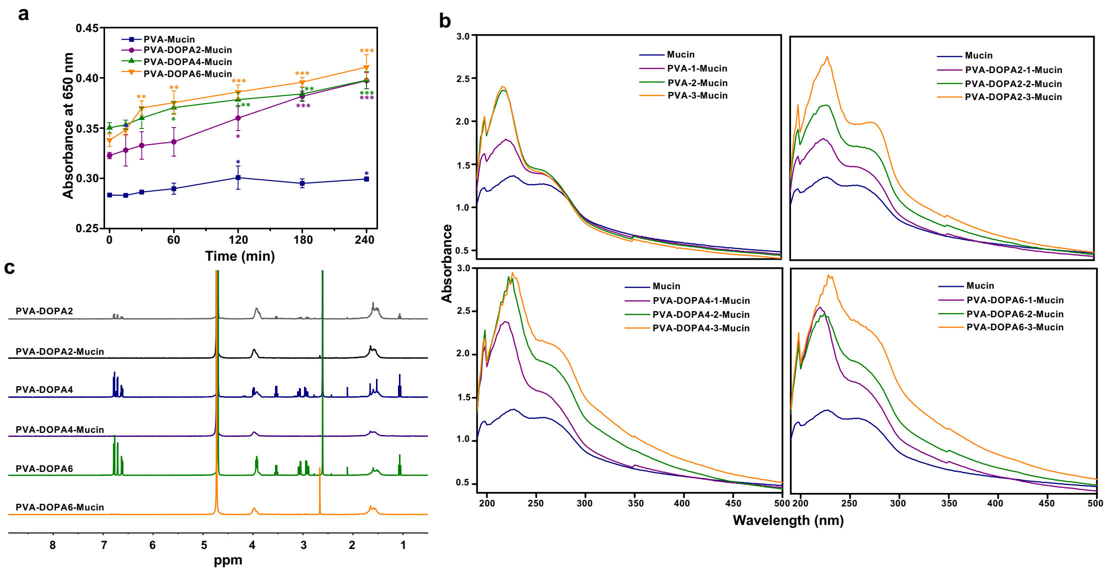

Supplementary Figure 3. Interactions of PVA-DOPA films with mucin. (a) Variation of turbidity of different PVA-DOPA-Mucin mixtures as a function of time. PVA: poly(vinyl alcohol), DOPA: 3,4-dihydroxy-D-phenylalanine. $n = 3$ independent samples per group; $*P < 0.05$; $**P < 0.01$; $***P < 0.001$ vs value at 0 h. All data are Mean ± S.D. Statistics was calculated by one-way ANOVA followed by Tukey's post-test. Exact P values are given in the Source Data file. (b) UV-vis absorbance spectra of different concentrations (0.05, 0.10, and 0.15 mg/ml) of PVA, PVA-DOPA2, PVA-DOPA4, and PVA-DOPA6 after mixed with mucin suspension. (c) Comparison of ^{1}H-NMR spectra of different PVA-DOPA polymers before and after mixed with mucin suspension. Source data are provided as a Source data file.

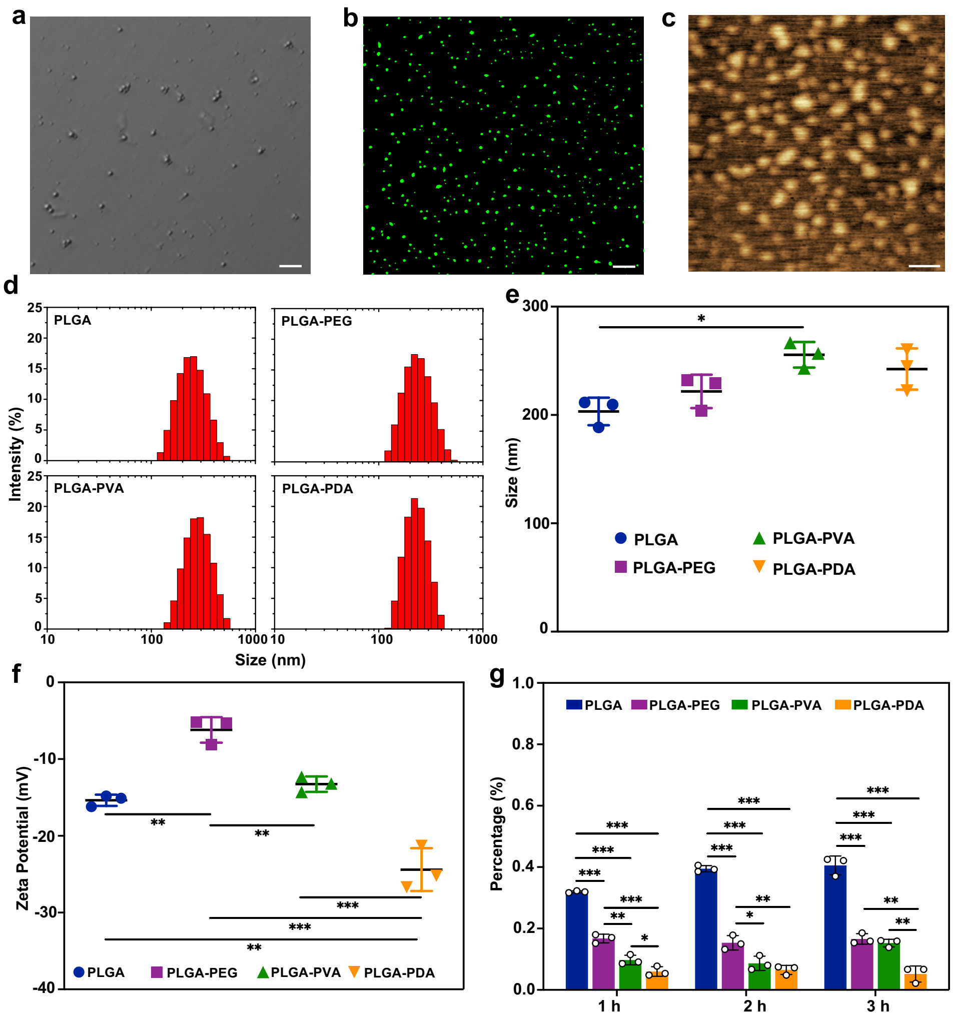

Supplementary Figure 4. Characterization of different PLGA NPs. (a) Optical micrography of PLGA NPs. Sale bar: 2 μm. (b) Fluorescence image of PLGA NPs. Sale bar: 2 μm. (c) AFM image of PLGA NPs. Sale bar: 200 nm. (d) Size distribution of PLGA, PLGA-PEG, PLGA-PVA, and PLGA-PDA NPs. PLGA: poly(lactic-co-glycolic acid), PEG: poly(ethylene glycol), PVA: poly(vinyl alcohol), PDA: polydopamine. (e) Particle size of PLGA, PLGA-PEG, PLGA-PVA, and PLGA-PDA NPs. $*P = 0.012$. (f) Zeta-potential of PLGA, PLGA-PEG, PLGA-PVA, and PLGA-PDA NPs. $**P < 0.01$; $***P < 0.001$. (g) Percentage of PLGA, PLGA-PEG, PLGA-PVA, and PLGA-PDA NPs interacted with rose bengal solution. $*P < 0.05$; $**P < 0.01$; $***P < 0.001$. All data are Mean ± S.D. $n = 3$ independent samples per group. Statistics was calculated by one-way ANOVA followed by Tukey's post-test. Exact P values are given in the Source Data file. Source data are provided as a Source data file.

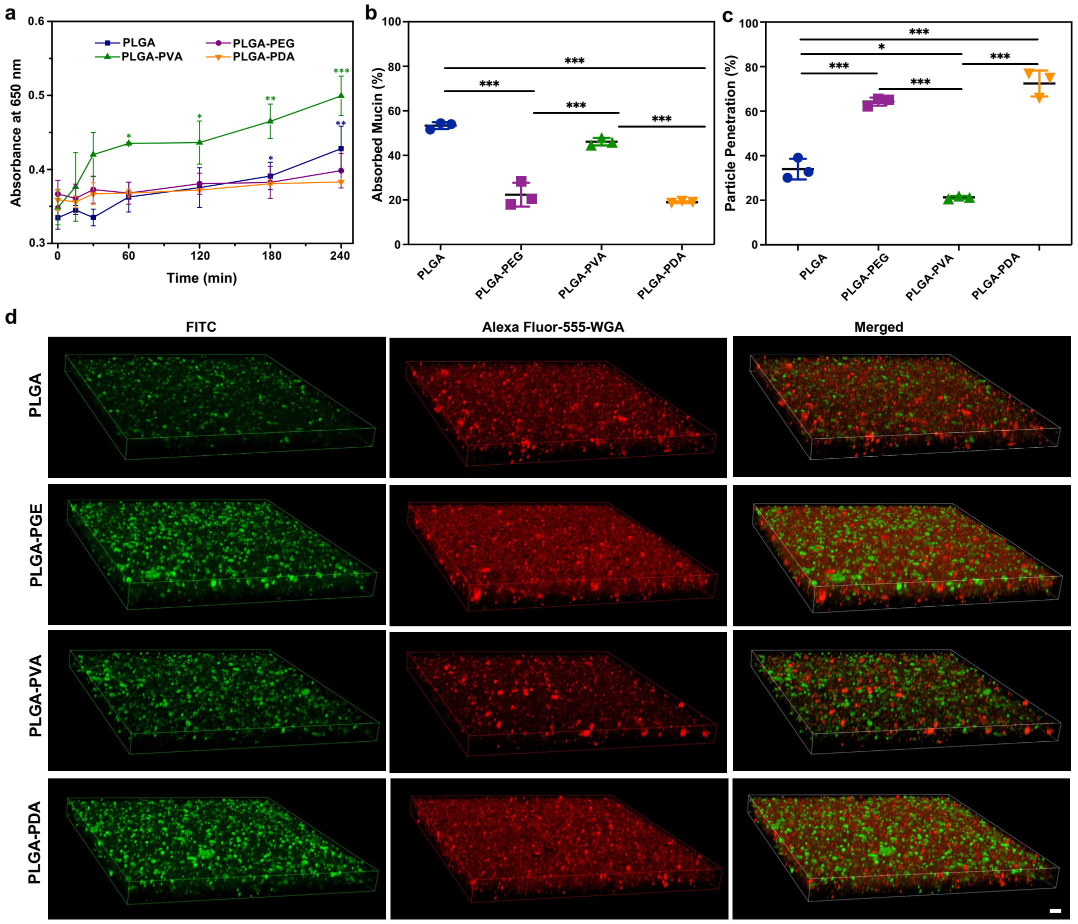

Supplementary Figure 5. Mucus-penetrating properties of different PLGA NPs. (a) Variation of turbidity of different NPs-Mucin mixtures as a function of time. PLGA: poly(lactic-co-glycolic acid), PEG: poly(ethylene glycol), PVA: poly(vinyl alcohol), PDA: polydopamine. *$P < 0.05$; **$P < 0.01$; ***$P < 0.001$ vs value at 0 h. (b) Percentage of absorbed PLGA, PLGA-PEG, PLGA-PVA, and PLGA-PDA NPs onto mucin particles. ***$P < 0.001$. (c) Percentage of penetrated NPs across mucus layer in an agarose gel assay after 6 h. *$P = 0.016$; ***$P < 0.001$ (d) *Z*-stacks of PLGA, PLGA-PEG, PLGA-PVA, and PLGA-PDA NPs diffusion (green) in mucin suspension (red). Scale bar: 50 µm. All data are Mean ± S.D. $n = 3$ independent samples per group. Statistics was calculated by one-way ANOVA followed by Tukey's post-test. Exact P values are given in the Source Data file. Source data are provided as a Source data file.

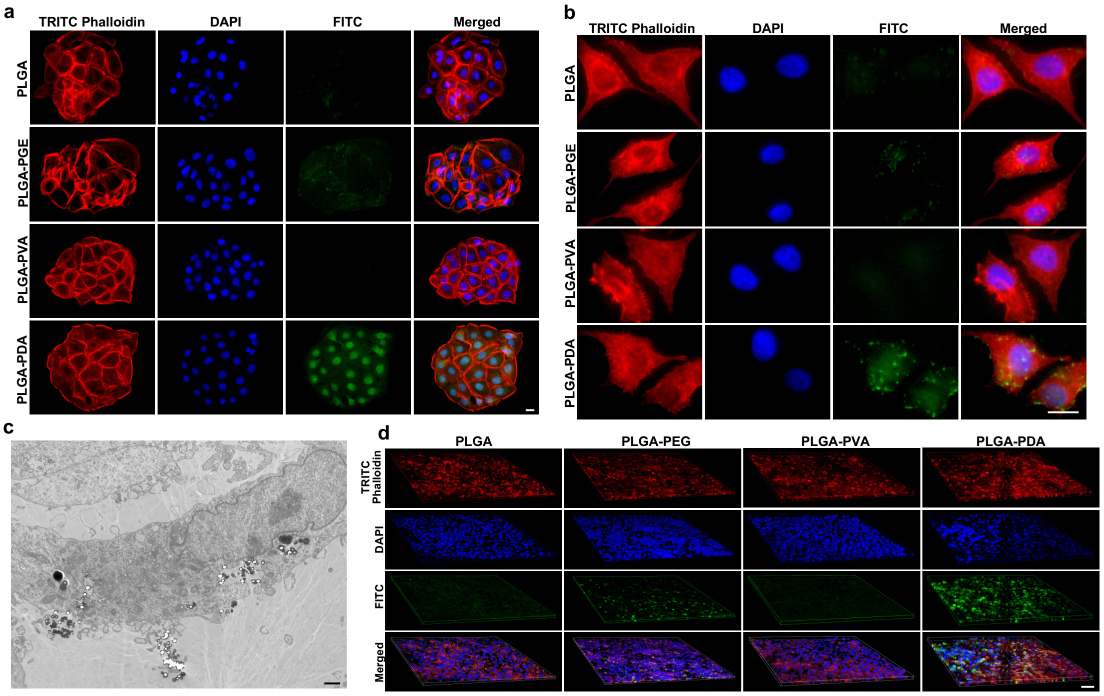

Supplementary Figure 6. Cellular uptake of different PLGA NPs in vitro. (a) Fluorescence image of cellular uptake of different NPs in HOK after incubation for 2 h. Scale bar: 20 µm. PLGA: poly(lactic-co-glycolic acid), PEG: poly(ethylene glycol), PVA: poly(vinyl alcohol), PDA: polydopamine. (b) Fluorescence image of cellular uptake of different NPs in HGECs after incubation for 2 h. Scale bar: 20 µm. (c)TEM images of cellular internalization process of PLGA-PDA NPs in HOK after incubation for 2 h. Scale bar: 1 µm. (d) 3D images of the cellular transport of NPs in the TR146 cell monolayer. Scale bar: 100 µm. *n* = 3 independent cells per group.

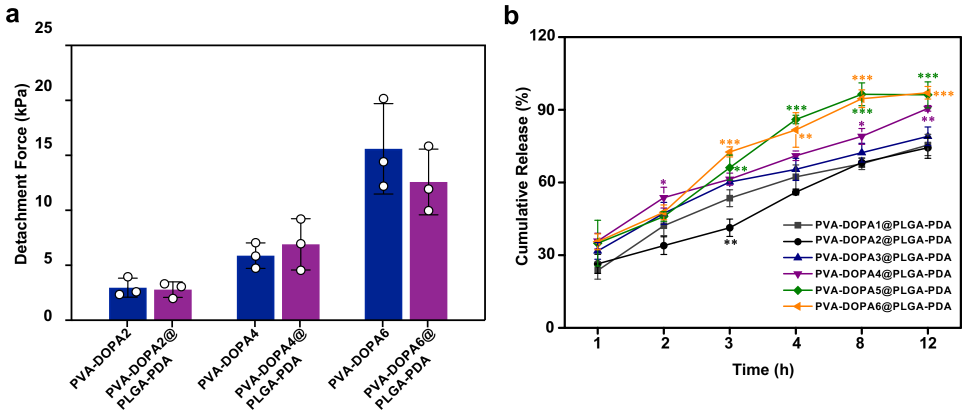

Supplementary Figure 7. Detachment force and release profile of PVA-DOPA@NPs films after incorporated with PLGA-PDA NPs. (a) Comparison of detachment force of different PVA-DOPA films before and after incorporated with NPs. PVA: poly(vinyl alcohol), DOPA: 3,4-dihydroxy-D-phenylalanine, PLGA: poly(lactic-co-glycolic acid), PDA: polydopamine. (b) Release profile of NPs from PVA-DOPA@PLGA-PDA films with different DOPA contents as a function of time. *$P < 0.05$; **$P < 0.01$; ***$P < 0.001$ vs PVA-DOPA1@PLGA-PDA group. All data are Mean ± S.D. $n = 3$ independent samples per group. Statistics was calculated by one-way ANOVA followed by Tukey's post-test. Exact P values are given in the Source Data file. Source data are provided as a Source data file.

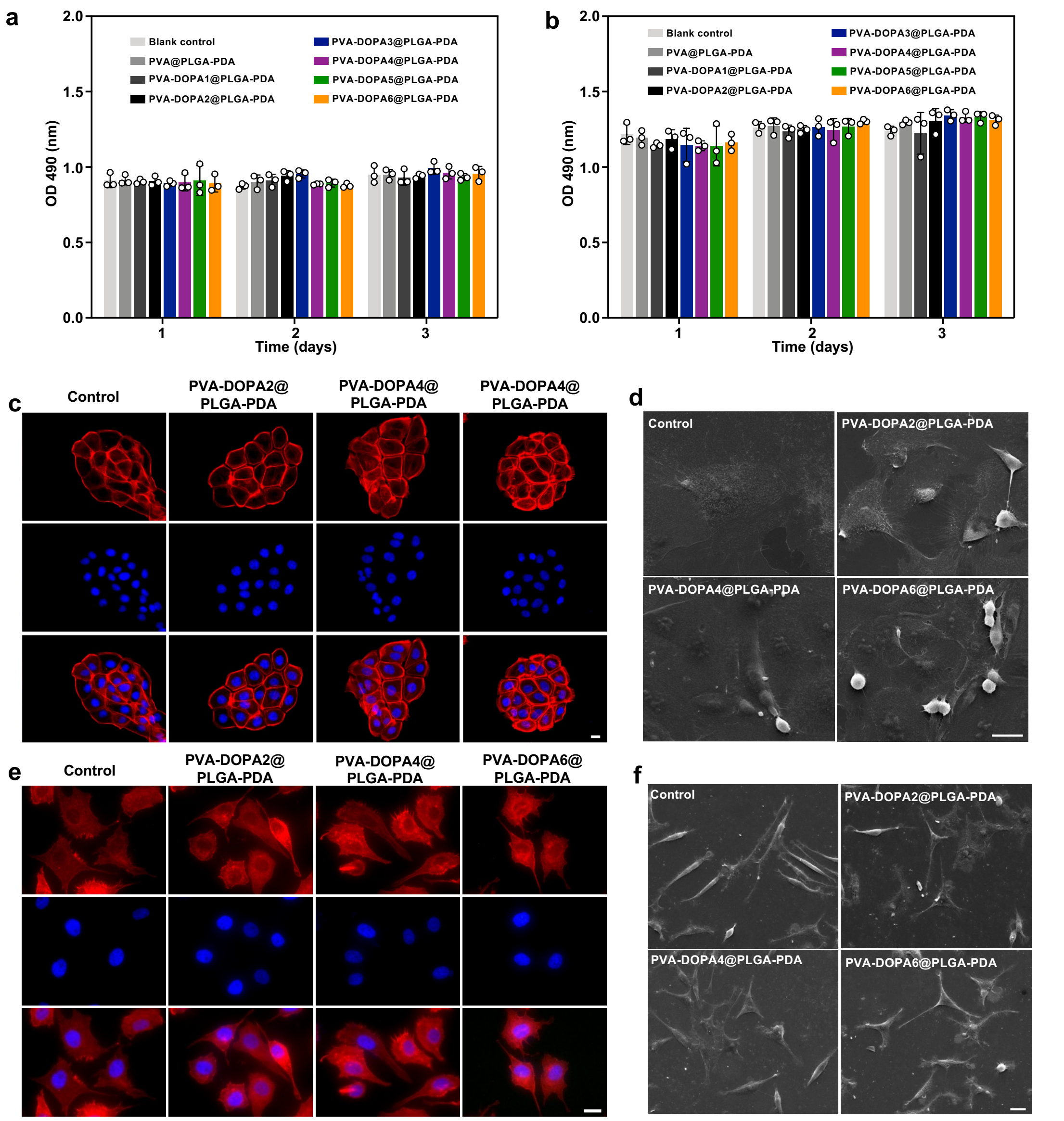

Supplementary Figure 8. In vitro biosafety evaluation of PVA-DOPA@PLGA-PDA film. (a) (b) CCK-8 assay of HOK and HGECs after incubated with different films for 1, 2, and 3 days, respectively. PVA: poly(vinyl alcohol), DOPA: 3,4-dihydroxy-D-phenylalanine, PLGA: poly(lactic-co-glycolic acid), PDA: polydopamine. (c) Fluorescence image of cell attachment of HOK after incubated with different PVA-DOPA@NPs films for 24 h. Scale bar: 20 μm. (d) SEM image of cell attachment of HOK after incubated with different PVA-DOPA@NPs films for 24 h. Scale bar: 20 μm. (e) Fluorescence image of cell attachment of HGECs after incubated with different PVA-DOPA@NPs films for 24 h. Scale bar: 20 μm. (f) SEM image of cell attachment of HGECs after incubated with different PVA-DOPA@NPs films for 24 h. Scale bar: 20 μm. All data are Mean ± S.D. $n = 3$ independent cells per group. Statistics was calculated by one-way ANOVA followed by Tukey's post-test. Source data are provided as a Source data file.

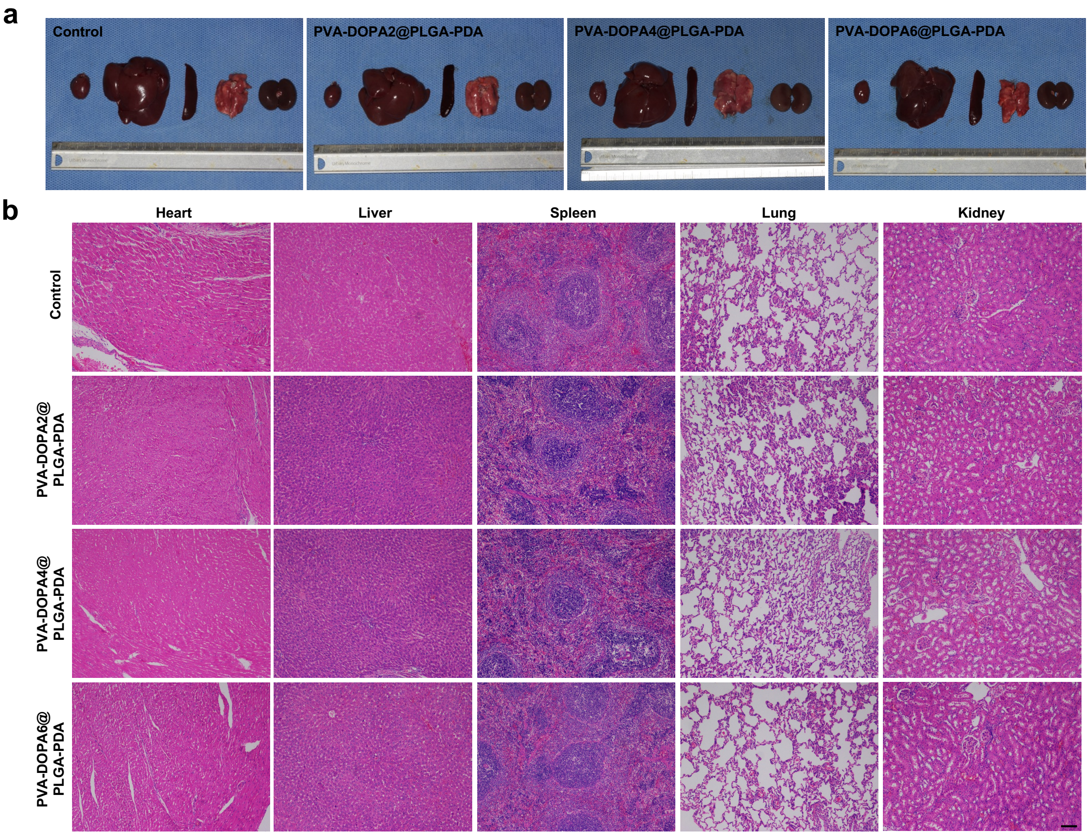

Supplementary Figure 9. In vivo biosafety evaluation of PVA-DOPA@PLGA-PDA film. (a) Photographs of rat major organs (heart, liver, spleen, lung, and kidney) after subcutaneously implanted with different films in the backs of SD rats for 7 days. PVA: poly(vinyl alcohol), DOPA: 3,4-dihydroxy-D-phenylalanine, PLGA: poly(lactic-co-glycolic acid), PDA: polydopamine. (b) H&E staining of major organs (heart, liver, spleen, lung, and kidney) after subcutaneously implanted with different films in the backs of SD rats for 7 days. Scale bars: 100 μm. *n* = 3 animals per group.

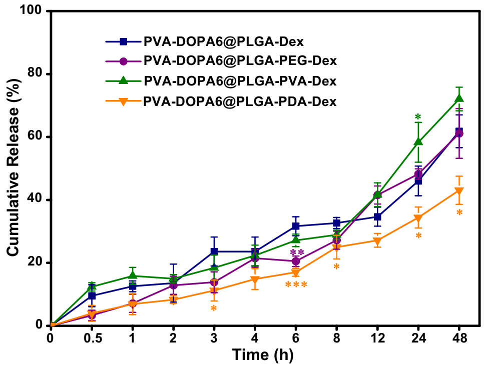

Supplementary Figure 10. In vitro release profile of Dex from PVA-DOPA6@NPs film incorporated with different NPs as a function of time. PVA: poly(vinyl alcohol), DOPA: 3,4-dihydroxy-D-phenylalanine, PLGA: poly(lactic-co-glycolic acid), PEG: poly(ethylene glycol), PVA: poly(vinyl alcohol), PDA: polydopamine. *$P < 0.05$; **$P = 0.001$; ***$P < 0.001$ vs PVA-DOPA6@PLGA-Dex group. All data are Mean ± S.D. $n = 3$ independent samples per group. Statistics was calculated by one-way ANOVA followed by Tukey's post-test. Exact P values are given in the Source Data file. Source data are provided as a Source data file.

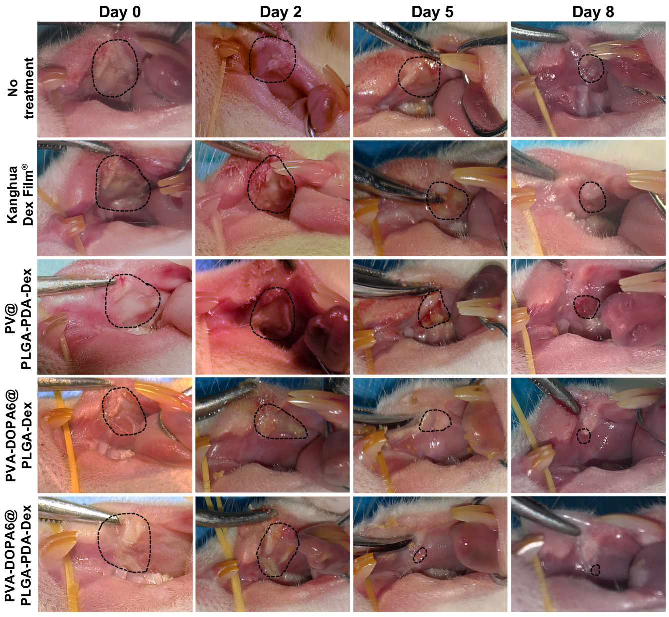

Supplementary Figure 11. Gross inspection of buccal mucosa ulcers in SD rats treated with Kanghua Dex Film®, PVA@PLGA-PDA-Dex, PVA-DOPA6@PLGA-Dex, PVA-DOPA6@PLGA-PDA-Dex film, and no treatment at day 0, 2, 5 and 8. Dex: dexamethasone, PVA: poly(vinyl alcohol), DOPA: 3,4-dihydroxy-D-phenylalanine, PLGA: poly(lactic-co-glycolic acid), PDA: polydopamine. $n = 3$ animals per group. (group 2)

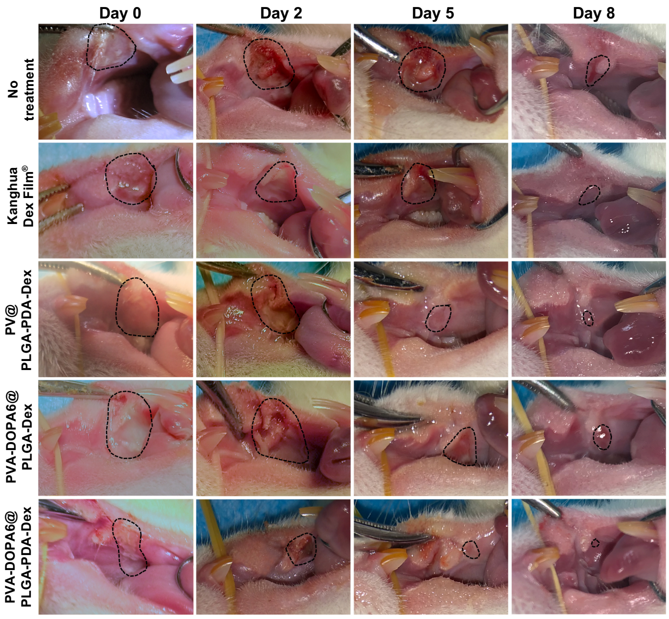

Supplementary Figure 12. Gross inspection of buccal mucosa ulcers in SD rats treated with Kanghua Dex Film®, PVA@PLGA-PDA-Dex, PVA-DOPA6@PLGA-Dex, PVA-DOPA6@PLGA-PDA-Dex film, and no treatment at day 0, 2, 5 and 8. Dex: dexamethasone, PVA: poly(vinyl alcohol), DOPA: 3,4-dihydroxy-D-phenylalanine, PLGA: poly(lactic-co-glycolic acid), PDA: polydopamine. $n = 3$ animals per group. (group 3)

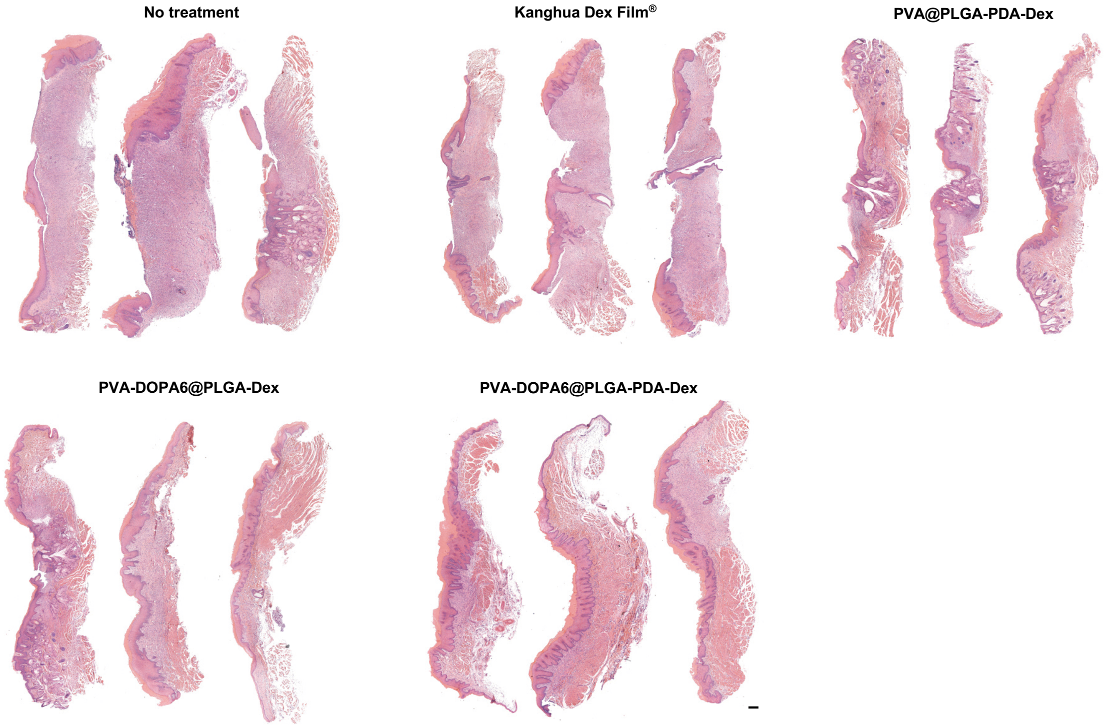

Supplementary Figure 13. H&E staining images of cross-section of regenerated oral ulcer in Sprague Dawley rats treated with Kanghua Dex Film®, PVA@PLGA-PDA-Dex, PVA-DOPA6@PLGA-Dex, PVA-DOPA6@PLGA-PDA-Dex film, and no treatment at day 8. Scale bar: 200 µm. $n = 3$ animals per group. (group 1-3)

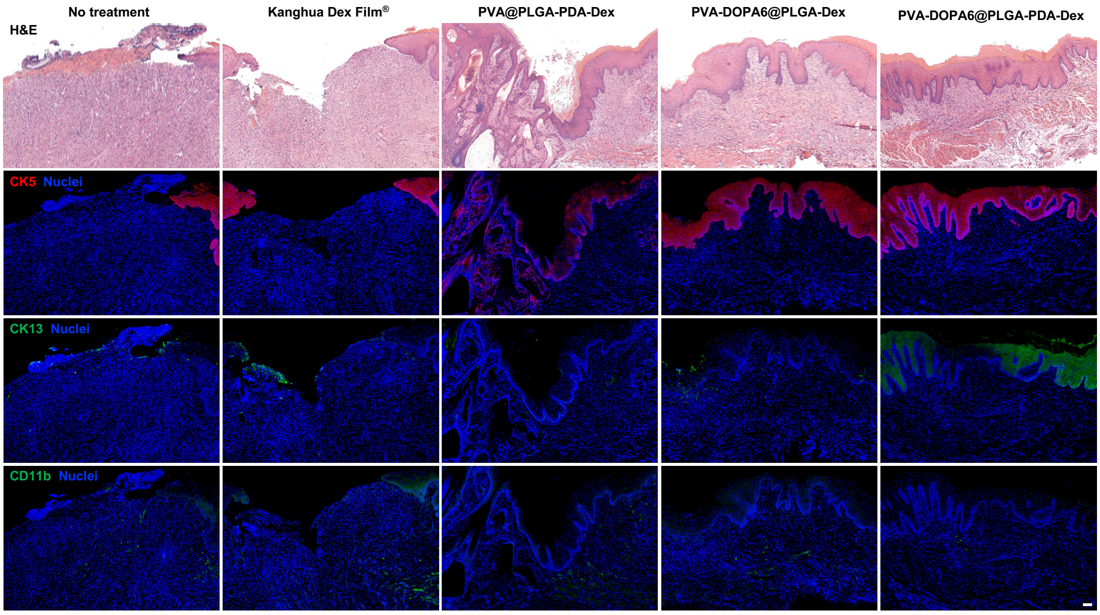

Supplementary Figure 14. H&E staining and immunohistochemistry staining of anti-keratin5 (CK5, red), anti-keratin13 (CK13, green), and anti-CD11b (CD11b, green) of regenerated oral ulcer at day 8. Nuclei (blue) was stained with DAPI. Scale bar: 100 μm. *n* = 3 animals per group. (group 2)

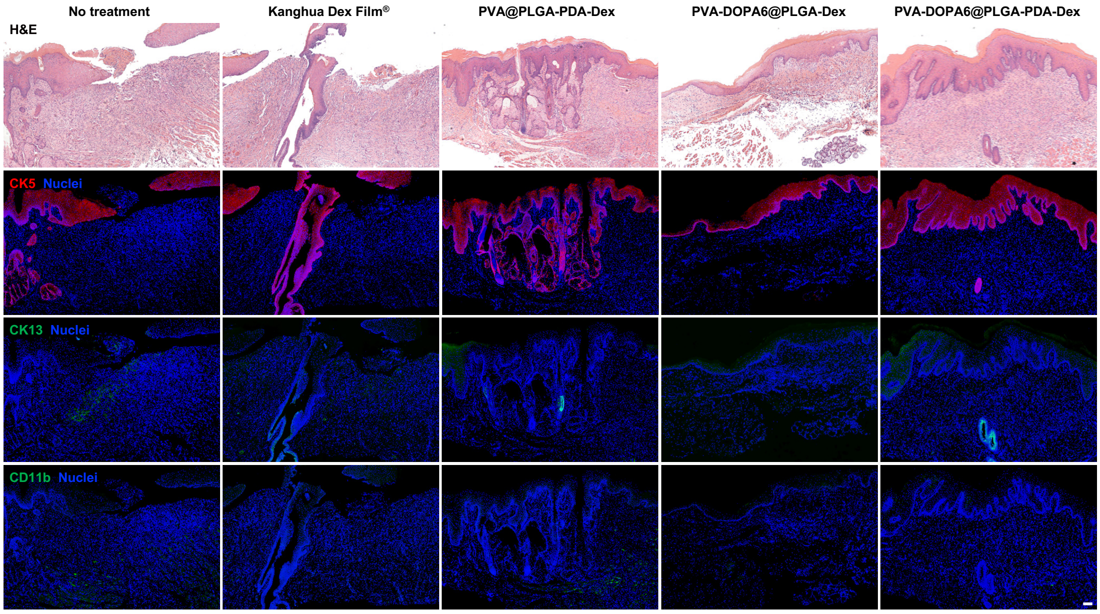

Supplementary Figure 15. H&E staining and immunohistochemistry staining of anti-keratin5 (CK5, red), anti-keratin13 (CK13, green), and anti-CD11b (CD11b, green) of regenerated oral ulcer at day 8. Nuclei (blue) was stained with DAPI. Scale bar: 100 μm. *n* = 3 animals per group. (group 3)

Supplementary Tables

Supplementary Table 1. Degree of substitution of catechol and mass fraction of catechol in PVA-DOPA conjugates calculated from the results of ^{1}H-NMR and UV-vis spectra.

Samples	Molar ratio of PVA/DOPA	Degree of substitution of catechol calculated from ^{1}H-NMR (%)	Mass fraction of catechol calculated from UV-vis (wt%)
PVA-DOPA1	6:1	4.7	16.0
PVA-DOPA2	6:2	9.3	27.9
PVA-DOPA3	6:3	28.0	35.0
PVA-DOPA4	6:4	41.3	42.2
PVA-DOPA5	6:5	57.3	61.5
PVA-DOPA6	6:6	64.6	72.0

Supplementary Table 2. Thickness and surface pH of different PVA-DOPA films. Data are presented as the means ± standard deviations (SDs). (n = 3 independent samples per group)

Samples	Thickness（mm）	Surface pH
PVA	1.06±0.03	6.8±0.06
PVA-DOPA1	1.04±0.03	6.6±0.08
PVA-DOPA2	1.00±0.04	6.6±0.05
PVA-DOPA3	0.99±0.05	6.7±0.05
PVA-DOPA4	1.00±0.06	6.7±0.08
PVA-DOPA5	1.04±0.06	6.6±0.08
PVA-DOPA6	1.03±0.06	6.7±0.12

Supplementary Table 3. Heat of fusion (ΔH_m) of different PVA-DOPA after interacted with mucin.

Samples	$\Delta H_m/Jg^{-1}$
PVA-Mucin	4.26
PVA-DOPA1-Mucin	26.80
PVA-DOPA2-Mucin	35.69
PVA-DOPA3-Mucin	46.09
PVA-DOPA4-Mucin	59.18
PVA-DOPA5-Mucin	73.58
PVA-DOPA6-Mucin	123.37

Supplementary Table 4. Pharmacokinetic parameters of Dex after administrated with different formulations of Dex via oral or buccal route in Sprague Dawley rats. Data are presented as the means ± standard deviations (SDs). (*n* = 3 animals)

**Samples**	$\boldsymbol{T_{max}}$ **(h)**	$\boldsymbol{C_{max}}$ **(ng/ml)**	$\boldsymbol{T_{1/2}}$ **(h)**	**AUC$_{0-24}$ (ng/ml*h)**
Dex (oral)	1.00	13.43±4.12	2.1±0.2	45.18±11.48
PLGA-PDA-Dex NPs (oral)	1.00	6.94±0.68	4.1±2.7	64.70±1.52
PVA-DOPA@PLGA-Dex film (buccal)	5.3±2.3	9.01±2.38	8.9±2.7	78.12±8.23
PVA-DOPA@PLGA-PDA-Dex film (buccal)	12.0	11.62±3.36	20.7±0.7	160.17±43.86

T_{max}: time at which C_{max} is attained; C_{max}: maximum plasma concentration; $T_{1/2}$: elimination half-life; AUC: area under concentration-time curve.
