# Supplementary material for: A mussel-inspired film for adhesion to wet buccal tissue and efficient buccal drug delivery
Source: Nat Commun. 2021 Mar 16;12:1689. doi: 10.1038/s41467-021-21989-5 (PMC7966365; doi:10.1038/s41467-021-21989-5)
Supplement: Supplementary file 3 — Description of Additional Supplementary Files [file 41467_2021_21989_MOESM3_ESM.pdf]

Description of Additional Supplementary Files

File Name: Supplementary Movie 1

Description: The shear strength of PVA-DOPA6 film on fresh porcine buccal tissue by lap-shear test using hands.

File Name: Supplementary Movie 2

Description: The shear strength of PVA-DOPA6 film on fresh porcine buccal tissue by lap-shear tests using a universal testing machine.

File Name: Supplementary Movie 3

Description: The tensile strength of PVA-DOPA6 film on fresh porcine buccal tissue by tensile tests using a universal testing machine.

File Name: Supplementary Movie 4

Description: The interfacial toughness of PVA-DOPA6 film on fresh porcine buccal tissue by peel tests using a universal testing machine.

File Name: Supplementary Movie 5

Description: The 3D mobility of PLGA-FITC NPs in mucus layer captured by super-resolution microscopy.

File Name: Supplementary Movie 6

Description: The 3D mobility of PLGA-PEG-FITC NPs in mucus layer captured by super-resolution microscopy.

File Name: Supplementary Movie 7

Description: The 3D mobility of PLGA-PVA-FITC NPs in mucus layer captured by super-resolution microscopy.

File Name: Supplementary Movie 8

Description: The 3D mobility of PLGA-PDA-FITC NPs in mucus layer captured by super-resolution microscopy.
